# Hemodynamic Predictors for Sepsis-Induced Acute Kidney Injury: A Preliminary Study

**DOI:** 10.3390/jcm9010151

**Published:** 2020-01-06

**Authors:** Oana Antal, Elena Ștefănescu, Monica Mleșnițe, Andrei Mihai Bălan, Alexandra Caziuc, Natalia Hagău

**Affiliations:** 1 Department of Anaesthesia and Intensive Care, “Iuliu Hațieganu” University of Medicine and Pharmacy, No 3-5 Clinicilor Street, Cluj-Napoca, 400005 Cluj, Romaniamonicamle@icloud.com (M.M.); balanandreimihai@yahoo.com (A.M.B.); alex.8610@gmail.com (A.C.); hagaunatalia@gmail.com (N.H.); 2 Department of Anaesthesia and Intensive Care, Cluj Emergency Clinical County Hospital, No 3-5 Clinicilor Street, Cluj-Napoca, 400005 Cluj, Romania

**Keywords:** sepsis-induced AKI, advanced hemodynamic monitoring

## Abstract

The aim of our study was to assess the association between the macrohemodynamic profile and sepsis induced acute kidney injury (AKI). We also investigated which minimally invasive hemodynamic parameters may help identify patients at risk for sepsis-AKI. We included 71 patients with sepsis and septic shock. We performed the initial fluid resuscitation using local protocols and continued to give fluids guided by the minimally invasive hemodynamic parameters. We assessed the hemodynamic status by transpulmonary thermodilution technique. Sequential organ failure assessment (SOFA score) (AUC 0.74, 95% CI 0.61–0.83, *p* < 0.01) and cardiovascular SOFA (AUC 0.73, 95% CI 0.61–0.83, *p* < 0.01) were found to be predictors for sepsis-induced AKI, with cut-off values of 9 and 3 points respectively. Persistent low stroke volume index (SVI) ≤ 32 mL/m^2^/beat (AUC 0.67, 95% CI 0.54–0.78, *p* < 0.05) and global end-diastolic index (GEDI) < 583 mL/m^2^ (AUC 0.67, 95% CI 0.54–0.78, *p* < 0.05) after the initial fluid resuscitation are predictive for oliguria/anuria at 24 h after study inclusion. The combination of higher vasopressor dependency index (VDI, calculated as the (dobutamine dose × 1 + dopamine dose × 1 + norepinephrine dose × 100 + vasopressin × 100 + epinephrine × 100)/MAP) and norepinephrine, lower systemic vascular resistance index (SVRI), and mean arterial blood pressure (MAP) levels, in the setting of normal preload parameters, showed a more severe vasoplegia. Severe vasoplegia in the first 24 h of sepsis is associated with a higher risk of sepsis induced AKI. The SOFA and cardiovascular SOFA scores may identify patients at risk for sepsis AKI. Persistent low SVI and GEDI values after the initial fluid resuscitation may predict renal outcome.

## 1. Introduction

Sepsis is still an important cause of morbidity and mortality in the intensive care unit (ICU) [1]. The combination of acute kidney injury (AKI) and sepsis carries an even higher mortality; sepsis-induced AKI was found to be a significant independent factor for mortality [2]. Sepsis is the leading cause of AKI in critically ill patients with a reported incidence of around 42.1% [3]. 

The pathophysiology of sepsis AKI is multifactorial, involving hemodynamic, microcirculatory, and inflammatory mechanisms [4]. Fluid management is a fundamental step in the management of this condition; it was already demonstrated that a successful goal-directed therapy decreases the risk of developing sepsis AKI [5]. 

Early identification and optimal management of patients at risk for sepsis AKI may lower the associated morbidity and mortality. The altered macrohemodynamic profile is one of the multiple triggers for sepsis induced AKI. The central role of the hemodynamic management in the prevention and treatment of patients with or at risk of sepsis AKI was already stated [6], but there is only limited research regarding the ability of the hemodynamic parameters in identifying the risk of AKI in the septic setting [7,8,9]. 

Advanced hemodynamic monitoring may be an essential tool in diagnosing the hemodynamic alterations and in achieving hemodynamic coherence [10,11]. Transpulmonary thermodilution technique was proven to be a reliable tool in assessing the hemodynamic status and in guiding fluid resuscitation in the critically ill [12,13]. By measuring cardiac output (CO) and its components (preload, afterload, and contractility) and by tailoring our interventions accordingly, we may improve diagnosis, treatment, and outcome. 

The aim of our study was to find advanced hemodynamic parameters that may help in the early identification of patients at risk of developing sepsis AKI.

## 2. Patients and Methods

This prospective observational study was carried out between 2016 and 2017, in a mixed surgical and medical ICU of a university hospital. The protocol was approved by the Ethics Committee of the University of Medicine and Pharmacy of Cluj-Napoca (no 119/6.03.2015). We obtained individual informed consent from each patient or from next of kin before data acquisition.

### 2.1. Study Patients

Seventy-one consecutive septic patients [14,15], recruited in the emergency department (ED) or hospital ward, were included in this study. Sepsis was defined as a life-threatening organ dysfunction caused by a dysregulated host response to infection, clinically defined as a qSOFA (quick sequential organ failure assessment) > 2, in the presence of suspected infection [15]. Organ dysfunction was defined as an acute change in total sequential organ failure assessment (SOFA) score of 2 points or greater secondary to infection [15]. Septic shock was defined by persisting hypotension requiring vasopressors to maintain a MAP of 65 mm Hg or higher and a serum lactate level greater than 2 mmol/L (18 mg/dL) despite adequate volume resuscitation [15].

All patients included in this study had no previous history of acute kidney disease or end-stage renal disease with oliguria or anuria, and had a normal urinary output prior to this hospital admission.

Patients were excluded if aged ≥80, previously known with cardiac failure NYHA III or IV, significant aortic valvular disease, severe pulmonary hypertension or cor pulmonale, hepatic failure, renal failure, known vascular disease, severe anaemia with no consent for red blood cells (RBCs) transfusion, or prone position. We used these complex exclusion criteria in order to avoid all factors that could bias the hemodynamics of the patients [16,17,18,19]. Both spontaneous breathing and mechanically ventilated patients were included in the study. 

### 2.2. Data Collection

Time zero (T_0_) was defined as the time of study inclusion in the intensive care unit (ICU). H_3_, H_6_, and H_24_ were defined as the 3rd, 6th, and 24th hour after study inclusion. Sepsis onset was defined as the moment when the patient with suspected infection met at least two points from the qSOFA or SOFA scores [15]; the time interval between sepsis onset and study inclusion time (T_0_) was less than two hours.

Fluid resuscitation in this time interval was carried out following local protocols (Supplemental Material 1A). Protocol compliance was achieved in all patients. 

From T_0_ to H_3_ fluid resuscitation was carried out according to the same local protocols (Supplemental Material 1A). Starting with the 3rd h (H_3_) to the 24th h (H_24_) after study inclusion, all patients continued to be resuscitated using minimally invasive hemodynamic monitoring parameters obtained through transpulmonary thermodilution techniques (EV1000, Edwards Lifesciences©, Irvine, CA, USA) and the local protocol (Supplemental Material 1B). Calibrations in the first 24 h were performed at H_3_, H_6_, and H_24_, and at any time the vasoactive infusion was adjusted. Static hemodynamic parameters and clinical features were also used in the monitoring process. Compliance to the fluid resuscitation protocol was achieved in all patients.

We used the vasopressor dependency index (VDI), to express the relationship between the vasopressor infusion dose and MAP. VDI is calculated as following: ((dobutamine dose × 1) + (dopamine dose × 1) + (norepinephrine dose × 100) + (vasopressin × 100) + (epinephrine × 100))/MAP [20]. Epinephrine, norepinephrine, dobutamine, and dopamine are expressed as µg/kg/min and vasopressin as units/min.

We defined vasoplegia as the syndrome of pathological low systemic vascular resistance, manifested clinically through the need for vasopressors in order to maintain a blood pressure ≥65 mm Hg in the absence of hypovolemia [21].

Sequential organ failure assessment (SOFA), cardiovascular SOFA, and acute physiology and chronic health evaluation (APACHE II) scores were used to classify the illness severity [22,23], while the kidney disease improving global outcomes (KDIGO) and acute kidney injury network (AKIN) urinary output criteria were used to define sepsis related AKI [24,25]. The rationale for choosing this clinical parameter at the expense of creatinine levels was due to the early and high sensitivity in predicting AKI [26]. 

We defined AKI as oliguria or anuria which persisted 24 h after sepsis diagnosis, after adequate fluid resuscitation was performed and obstruction was ruled out [25].

According to the renal outcome at 24 h, we separated the patients in two groups: the oliguric/anuric group, 19 patients (oliguric/anuric patients at 24 h after enrollment) and the normal urinary output group, 49 patients (patients which were with normal diuresis both at the time of study inclusion and 24 h later and the patients which were initially oliguric/anuric but restored normal diuresis by the 24th h after enrollment). This stratification was performed after the exclusion of patients with mortality < 24 h and patients with continuous renal replacement therapies (CRRT).

### 2.3. Statistical Analysis

For the statistical analysis we used IBM SPSS Statistics (version 23.0, IBM Corp, Armonk, NY, USA) MedCalc statistical software (version 17.9, MedCalc Software, Ostend, Belgium) Microsoft Excel (2013, Microsoft Corporation, Redmond, WA, USA), and GraphPad Prism (6, GraphPad Software, La Jolla, CA, USA). Continuous variables were expressed as mean ± SD and categorical variables as numbers or percentages. For descriptive statistics we used tables and graphs. To compare means we used the Wilcoxon signed rank test and Mann–Whitney U test and independent samples t-test. Proportions were compared using the two-proportion Z-Test; Receiver operating characteristic (ROC) curve analysis was used to determine predicting factors and cutoff points; odds ratio (OR) and relative risk (RR) were used as measures of association; a *p* < 0.05 was considered to be statistically significant.

## 3. Results

All 71 patients were included in the statistical analysis. Their demographic and physiologic characteristics are shown in Table 1.

By the 3rd h after study inclusion (H_3_) most of the macro hemodynamic parameters were in the targeted range (Supplemental Material 2). An improvement in microcirculation was also noted, as shown by the reduction in the number of patients with increased capillary refill time (CRT, *p* < 0.05 at 6 hours and *p* < 0.01 at 24 h), the reduction in the number of patients presenting oliguria/anuria (*p* < 0.05 at H_3_ and *p* < 0.01 at H_6_ and H_24_), and the reduction in serum lactate level (for the septic shock patients, *p* < 0.0001 at H_24_) (Supplemental Material 2). We considered the fluid resuscitation to be appropriate as we noticed an improvement in these macro- and micro-hemodynamic parameters. 

The incidence of sepsis induced AKI in our study was 27.9%, as shown by the number of oliguric/anuric vs. normal urinary output patients (19 vs. 49).

When we compared the SOFA and cardiovascular SOFA scores at T_0_ among the two groups we found statistically significant differences (*p* < 0.05). 

**Figure 1:**
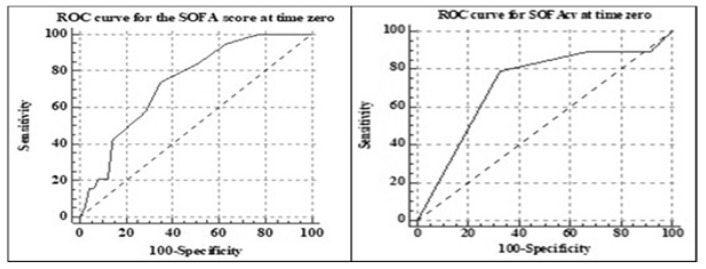
Receiver operating characteristic (ROC) curve analysis for Sequential organ failure assessment (SOFA) and cardiovascular SOFA at time zero.

The ROC curve analysis for the SOFA score identified a cutoff point of >9 points (AUC 0.74, SE 0.06, 95% CI 0.61–0.83, *p* < 0.01) and a cutoff point of >3 for the cardiovascular SOFA for identifying patients at risk of oliguria/anuria (AUC 0.73, SE 0.06, 95% CI 0.61–0.83, *p* < 0.01). The graphical representation is shown in Figure 1.

**Figure 2:**
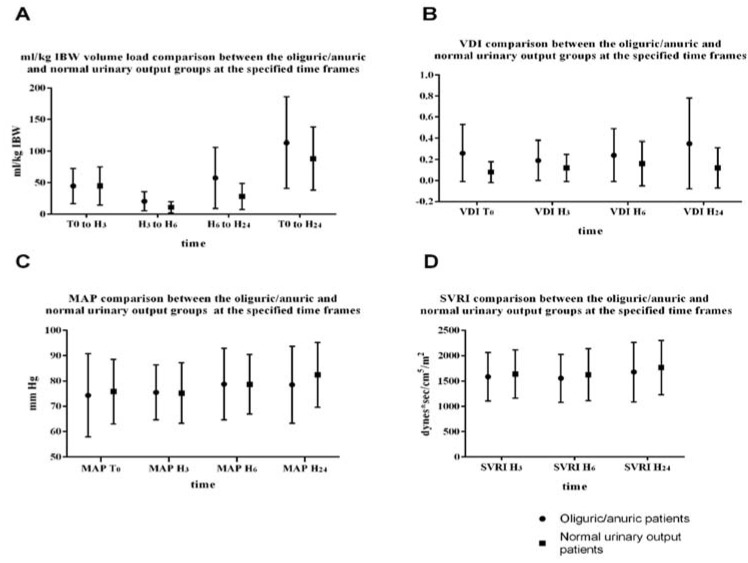
Comparison between the poor and normal urinary output groups at the specified time frames. (**A**) Fluid load, (**B**) vasopressor dependency index, (**C**) mean arterial blood pressure, (**D**) systemic vascular resistance index.

If we compare the total fluid load (from T_0_ to the H_24_) among the two groups, we can observe that the anuric/oliguric patients received more fluids compared to the normal urinary output group (113.43 ± 72.73 versus 88.02 ± 50.06 (Figure 2A). 

**Figure 3:**
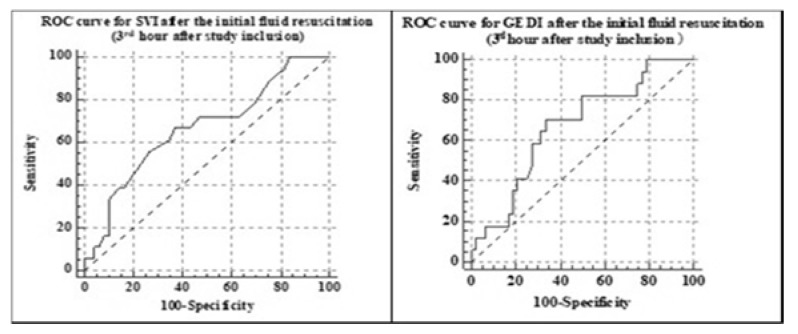
ROC curve analysis for the stroke volume index (SVI) and global end diastolic index (GEDI) after the initial fluid resuscitation.

From T_0_ to H_3_ the fluid load was similar among the two groups (Figure 2A). Still the 3rd h minimally invasive hemodynamic evaluation showed a statistically significant lower stroke volume index (31.5 ± 9.3 compared to 37.0 ± 9.6, *p* = 0.03) and global end diastolic index (565.8 ± 133.6 versus 661.8 ± 158.4, *p* = 0.03) in the oliguric/anuric group compared to the normal urinary output group. The ROC curve analysis showed a cutoff point of 32 mL/m^2^/beat for SVI (AUC 0.67, SE 0.07, 95% CI 0.54–0.78, *p* < 0.05) and a cutoff value of 583 mL/m^2^ for GEDI (AUC 0.67, SE 0.07, 95% CI 0.54–0.78, *p* < 0.05) as predictive for oliguria/anuria at 24 h after study inclusion (Figure 3). There were no statistically significant differences in the MAP and SVRI among the two groups, even though the patients in the oliguric/anuric group had statistically significant more norepinephrine (*p* < 0.001, at T_0_, and *p* < 0.02 at H_24_, Table 2) and statistically significant higher VDI levels (*p* < 0.001, at T_0_, and *p* < 0.01 at H_24_, Table 2). Both the difference in norepinephrine infusion and the higher VDI were suggestive for a more severe vasoplegia in the oliguric/anuric. 

Patients who had an SVI lower than the cutoff value had a higher risk of remaining oliguric/anuric at 24 h than the patients with normal SVI (OR = 3.44, 95% CI 1.1–10.76, *p* = 0.03); the calculated relative risk (RR) was 2.46 (CI 1.05–5.79, *p* = 0.03). 

The renal outcome upon discharge or at 28 days after admission showed a higher creatinine level in the anuric/oliguric group compared to the normal urinary output group (164.4 µmol/L ± 255.1 vs. 95.4 µmol/L ± 70.0, *p* = 0.14). The number of ICU days among the two groups showed no statistically significant difference (19.3 ± 14.4 in the oliguric/anuric group compared to 17.4 ± 13.9 in the normal urinary output group, *p* = 0.70).

The all-cause mortality for all patients included in the study was 30.9%. The all-cause mortality within the normal urinary output patients was 22.4%, while in the oliguric/anuric patients was 52.63%. The odds ratio was 3.84, 95% CI 1.24–11.80, *p* = 0.01; the RR was 2.43, 95% CI 1.19–4.59, *p* = 0.01.

## 4. Discussion

The main finding of our study is the fact that renal outcome in patients with sepsis and septic shock may be predicted by severe vasoplegia in the first 24 h of sepsis. A persistent low SVI (≤32 mL/m^2^/beat) and low GEDI (<583 mL/kg) after the initial fluid resuscitation are also predictive for sepsis AKI. There are few studies which investigate the relationship between hemodynamics and progression of AKI during early phases of sepsis, and, from our knowledge, there are no studies which focus on the predicting value of vasoplegia, SVI or GEDI [7,8]. 

The cutoff values found on the ROC curves analysis for the stroke volume index and global end-diastolic volume are lower than the normal values specified by the manufacturer. The association between a low SVI and a low GEDI is suggestive for a low preload. Therefore, we may argue that the patients in the oliguric/anuric group did not receive enough fluids. But as shown in Figure 2A, not only they received similar amounts of fluids in the initial fluid resuscitation, but in the next few hours, they were given more fluids, in the attempt to restore normal GEDI, SVI, and urinary output. If we add the fact that patients in the oliguric/anuric group were having both statistically higher VDI and norepinephrine infusion rates to maintain the SVRI in clinically acceptable ranges (Table 2), and also a significantly higher pulmonary SOFA at time 0 (*p* < 0.05, Table 2), we may state that these patients were having a more severe vasoplegia with both enhanced vascular compliance and capillary leakage [27,28]. This group of patients has a high risk for fluid overload, a status associated with increased mortality in sepsis. 

A high SOFA score (>9 points) and a high cardiovascular SOFA (>3 points) at time zero may be predictors for sepsis AKI. The cutoff values found for the SOFA and cardiovascular SOFA may represent tools for screening the septic patients at risk for AKI. The ease in obtaining these scores made them efficient screening methods for sepsis and septic complications [15,29]. 

We used only the urine output criterion to define AKI at 24 h after study inclusion. The rationale for choosing this clinical parameter at the expense of creatinine levels was due to the early and high sensitivity in predicting AKI [26]. Kellum et al. demonstrated that AKI defined by isolated oliguria (no SC criteria present) was surprisingly frequent and was associated with a long-term morbidity and mortality [30]. In their study they also emphasized that some of the critically ill patients may have fluid overload with impact on the measured serum creatinine levels [30]. The mean values of creatinine among the two groups at admission and 24 h later (as shown in Table 2) are higher in the oliguric/anuric group compared to the normal urinary output group, but don’t show statistically significant differences. 

The incidence of sepsis induced AKI found in our study was lower compared to other studies [2,5,31]. This could be since we stratified the patients according to the 24 h renal outcome, including only patients with stage 2 and 3 AKIN and KDIGO acute kidney injury scores. This could be a limitation of our study.

All-cause mortality for the patients included in the study was 30.9%, similar to the one found in other significant research on the subject [3,32]. AKI is known to be an independent risk factor for in-hospital mortality [2]. Our research showed that patients which developed AKI had twice the mortality rate of septic patients without AKI, in concordance with other important works [33,34]. A possible limitation in our study was the fact that we did not calculate mortality with adjustments for SOFA or APACHE II scores. 

The renal outcome at 28 day or upon discharge among survivors was similar in the two studied groups, but a larger study is needed in order to confirm these findings. The number of ICU days among the two groups showed no statistically significant difference (19.3 ± 14.4 in the oliguric/anuric group compared to 17.4 ± 13.9 in the normal urinary output group, *p* = 0.70), but due to the small sample size, further research is needed to confirm this result.

Our study has several limitations. Due to the complex exclusion criteria, which had the purpose of reducing the bias generated by the hemodynamic monitoring (e.g., severe valvular diseases may impair the results of the transpulmonary thermodilution hemodynamic monitoring parameters), our results cannot be extrapolated to all septic patients. Moreover, the sample size was also limited, and the study is underpowered; more research is needed in order to confirm these results. 

The lack of temporal relationship as AKI onset after sepsis onset is probably the biggest weakness in research method.

Another limitation of our study is related to the ROC AUC values which are modest, especially in the context of the multiple factors involved in the onset and persistence of oliguria and sepsis related AKI. Furthermore, the differences in baseline characteristics and number of patients in the two groups are possible factors for further errors. The results obtained through a case control experimental design, matched for selected baseline factors, could support the results obtained in this observational study; further research is needed.

## 5. Conclusions

Severe vasoplegia in the first 24 h of sepsis is associated with a higher risk of sepsis induced AKI. The SOFA and cardiovascular SOFA may help identify patients at risk for sepsis-induced AKI. Renal outcome in patients with sepsis and septic shock may be predicted by a persistent low SVI (≤32 nmL/m^2^/beat) and low GEDI (<583 mL/kg) after the initial fluid resuscitation. Further research is needed to confirm these results.

## Figures and Tables

**Table 1:** Clinical and demographic characteristics of the patients included in the study.

	All Patients Included in the Study	Oliguric/Anuric Group	Normal Urinary Output Group	*p* Value *
Number of patients N	71	19	52	
Age Mean ± SD	62.6 ± 14.7	61.4 ± 10.7	62.9 ± 15.1	0.57
Weight (actual) kg Mean ± SD	82.5 ± 20.0	88.5 ± 21.4	79.9 ± 19.6	0.14
Body Surface Area Mean ± SD	1.9 ± 0.2	2.0 ± 0.2	1.9 ± 0.2	0.09
Diagnosis N (%)				
Sepsis	37 (52.1)	13 (68.4)	21 (40.4)	0.03
Septic shock	34 (47.9)	6 (31.6)	31 (59.6)	0.03
Type of sepsis N (%)	N (%)			
Medical	26 (36.6)	8 (42.1)	19 (36.5)	0.66
Surgical	45 (63.4)	11 (57.9)	33 (63.5)	0.66
Ventilation N (%)	N (%)			
Mechanically ventilated	49 (69)	18 (94.7)	31 (59.6)	0.04
Spontaneous ventilation	22 (31)	1 (5.3)	21 (40.4)	0.04
PEEP for Mechanically ventilated at study inclusion (T_0_) Mean ± SD	5.7 ± 1.1	6 ± 1.2	5.5 ± 0.9	0.16
SOFA Score at study inclusion (T_0_) Mean ± SD points	9.5 ± 3.2	11.3 ± 2.9	8.8 ± 3.2	0.02
SOFA Score without renal SOFA at study inclusion (T_0_) Mean ± SD points	7.1 ± 2.5	8.2 ± 2.2	6.8 ± 2.6	0.06
Cardiovascular SOFA at study inclusion (T_0_) Mean ± SD points	2.8 ± 1.4	3.4 ± 1.2	2.5 ± 1.3	0.03
APACHE II Score at study inclusion (T_0_) Mean ± SD points	21.9 ± 8.6	23.3 ± 8.5	20.8 ± 8.4	0.17
Heart Rate at study inclusion (T_0_) Mean ± SD beats/min	105.0 ± 20.6	108.5 ± 19.2	101.6 ± 18.0	0.15
Mean arterial blood pressure (MAP) at study inclusion (T_0_) Mean ± SD mm Hg	75.2 ± 13.6	74.3 ± 16.4	75.8 ± 12.8	0.68
Lactate at study inclusion (T_0_) Mean ± SD mmol/l	2.52 ± 2.2	4.1 ± 2.0	3.5±2.3	0.12
Norepinephrine at study inclusion (T_0_) Mean ± SD mcg/kg/min	0.09 ± 0.1	0.18 ± 0.1	0.06 ± 0.07	0.001
VDI Mean ± SD at study inclusion (T_0_)	0.14 ± 0.2	0.26 ± 0.27	0.08 ± 0.1	0.001
Creatinine Mean ± SD at study inclusion (T_0_) µmol/l	218.3 ± 192.7	291.7 ± 226.3	192.76 ± 179.5	0.06
Urea Mean ± SD at study inclusion (T_0_) mmol/l	16.4 ± 12.0	20.3 ± 13.7	14.6 ± 11.2	0.09

* *p* value between the oliguric/anuric group and the normal urinary output group.

**Table 2:** The hemodynamic parameters of the two groups of patients.

	T_0_			H_3_			H_6_			H_24_		
	Oliguric/Anuric Group	Normal Urinary Output Group	*p* Value	Oliguric/Anuric Group	Normal Urinary Output Group	*p* Value	Oliguric/Anuric Group	Normal Urinary Output Group	*p* Value	Oliguric/Anuric Group	Normal Urinary Output Group	*p* Value
SOFA Mean ± SD points	11.3 ± 2.9	8.8 ± 3.2	0.02	not calculated at the 3rd h			10.1 ± 3.1	8.4 ± 3.6	0.11	10.0 ± 2.5	7.2 ± 3.6	0.02
SOFAcv Mean ± SD points	3.4 ± 1.2	2.5 ± 1.3	0.03				3.0 ± 1.6	2.8 ± 1.5	0.22	3.0 ± 1.6	2.3 ± 1.5	0.03
SOFAr Mean ± SD points	3.1 ± 1.4	1.9 ± 1.6	0.03				2.8 ± 1.5	1.3 ± 1.5	0.01	2.7 ± 1.3	1.1 ± 1.3	0.00
SOFAp Mean ± SD points	2.4 ± 1.2	1.8 ± 1.2	0.02				2.2 ± 1.2	1.0 ± 1.0	0.18	2.1 ± 0.9	1.6 ± 1.0	0.12
APACHE II Mean ± SD points	23.3 ± 8.5	20.8 ± 8.4	0.17				not calculated at 6th h			not calculated at 24th h		
SBD Mean ± SD mm Hg	121.1 ± 23.9	117.5 ± 19.9	0.53	124.2 ± 18.5	123.0 ± 17.9	0.80	126.1 ± 17.3	127.9 ± 18.5	0.70	130.5 ± 17.9	130.1 ± 20.0	0.93
DBP Mean ± SD mm Hg	57.7 ± 14.5	55.6 ± 10.0	0.67	58.0 ± 11.9	55.0 ± 11.9	0.34	60.5 ± 12.0	56.5 ± 11.7	0.21	60.4 ± 12.3	59.5 ± 13.3	0.80
MAP Mean ± SD mm Hg	74.3 ± 16.4	75.8 ± 12.8	0.68	75.4 ± 10.9	75.2 ± 11.9	0.94	78.7 ± 14.1	78.6 ± 11.7	0.97	78.5 ± 15.2	82.4 ± 12.7	0.29
Heart rate Mean ± SD beats/min	108.5 ± 19.2	101.5±17.9	0.15	101.7 ± 18.7	96.5 ± 18.3	0.26	100.1 ± 21.0	98.1 ± 19.5	0.90	101.1 ± 14.9	95.5±17.4	0.25
CVP Mean ± SD mm Hg	8.5 ± 4.2	6.8 ± 4.7	0.08	10.7 ± 3.9	7.6 ± 4.9	0.006	11.2 ± 3.7	8.2 ± 4.9	0.01	7.7 ± 3.9	8.3 ± 4.6	0.96
CI Mean ± SD l/min	not monitored at time 0			3.2 ± 0.8	3.5 ± 0.9	0.20	3.1 ± 0.8	3.7 ± 0.9	0.03	3.2 ± 0.6	3.5 ± 0.7	0.23
SVI Mean ± SD mL/m^2^/beat				31.5 ± 9.4	37.0 ± 9.6	0.03	34.1 ± 12.2	38.0 ± 10.0	0.27	33.1 ± 8.6	38.0 ± 10.4	0.07
GEDI Mean ± SD mL/kg				565.8 ± 133.6	661.8 ± 158.4	0.037	530.9 ± 199.3	651.9 ± 203.0	0.07	605.1 ± 120.6	707.4 ± 153.6	0.009
ITBI Mean ± SD mL/m^2^				754.5 ± 215.6	764.6 ± 153.0	0.15	761.9 ± 247.0	858.3 ± 262.2	0.20	776.4 ± 247.8	931.4 ± 229.8	0.009
ELWI Mean ± SD mL/kg				7.9 ± 2.0	8.7 ± 3.3	0.88	8.88 ± 3.03	8.9 ± 4.0	0.49	8.8 ± 2.2	8.5 ± 3.0	0.45
GEF Mean ± SD				23.6 ± 8.7	22.3 ± 5.8	0.91	23.6 ± 9.8	22.7 ± 6.2	0.96	22.7 ± 7.0	21.1 ± 6.2	0.64
SVRI Mean ± SD dynes * sec/cm^5^/m^2^				1584.4 ± 477.4	1638.9 ± 476.4	0.97	1554.3 ± 472.3	1623.7 ± 512.1	0.85	1678.0 ± 588.0	1765.6 ± 536.0	0.69
Norepinephrine Mean ± SD mcg/kg/min	0.18 ± 0.19	0.06 ± 0.07	0.001	0.14 ± 0.14	0.08 ± 0.08	0.08	0.17 ± 0.16	0.10 ± 0.12	0.11	0.24 ± 0.30	0.12 ± 0.19	0.02
VDI Mean ± SD	0.26 ± 0.27	0.08 ± 0.1	0.001	0.19 ± 0.19	0.12 ± 0.13	0.14	0.24 ± 0.25	0.16 ± 0.21	0.19	0.35 ± 0.43	0.12 ± 0.19	0.01
Creatinine Mean ± SD) µmol/L	291.7 ± 226.3	192.76 ± 179.5	0.06	not monitored at these time frames						249.3 ± 191.8	184.8 ± 165.3	0.14
Urea Mean ± SD mmol/L	20.32 ± 13.7	14.6 ± 11.28	0.09							19.0 ± 10.7	15.3 ± 11.6	0.11
Mean urinary output Mean ± SD mL/kg/hour										0.12 ± 0.12	1.26 ± 0.75	<0.001
Lactate (septic shock patients) mean ± SD mmol/L	4.1 ± 2.0	3.5 ± 2.3	0.12	3.6 ± 2.0	3.6 ± 3.4	0.08	3.5 ± 1.5	3.7 ± 3.4	0.18	2.2 ± 1.2	2.5 ± 2.7	0.18
Lactate clearance ≥ 10% (septic shock patients) %	not monitored between time of presentation and time zero			53.8	44.4	0.60	53.8	44.4	0.60	84.6	94.4	0.36
Capilary refill time > 3 sec %	31.60	16.30	0.16	26.30	10.10	0.09	21.10	6.2%	0.06	5.30	4.10	0.08

T_0_: time zero, time of study inclusion; H_3_, H_6_, H_24_: 3rd, 6th, and 24th h transpulmonary thermodilution calibrations performed in Ev1000. SOFAcv: cardiovascular SOFA; SOFAr: renal SOFA; SOFAp: pulmonary SOFA; SBD: systolic blood pressure; DBP: dyastolic blood pressure; MAP: mean arterial blood pressure; CVP: central venous pressure; CI: cardiac index; SVI: stroke volume index; GEDI: global end-dyastolic index; ITBI: intrathoracic blood index; ELWI: extravscular lung water index; GEF: global ejection fraction; SVRI: systemic vascular resistance index; VDI: vasopressor dependency index.

## Supplementary Materials

The following are available online at https://www.mdpi.com/2077-0383/9/1/151/s1, Supplemental Material 1A: Fluid resuscitation protocol in the absence of advanced hemodynamic monitoring, Supplemental Material 1B: Fluid resuscitation protocol guided by advanced hemodynamic monitori, Supplemental Material 2: The hemodynamic parameters of the patients included in the study.
