# Supplementary material for: Hemodynamic Predictors for Sepsis-Induced Acute Kidney Injury: A Preliminary Study
Source: J Clin Med. 2020 Jan 6;9(1):151. doi: 10.3390/jcm9010151 (PMC7019750; doi:10.3390/jcm9010151)
Supplement: Supplementary file 1 [file jcm-09-00151-s001.pdf]

A. Fluid resuscitation protocol in the absence of advanced hemodynamic monitoring

20-30 mL/kg crystalloids initial fluid load
PPV >12% at PLR, additional 250 mL fluid bolus was administered
CVP > 12 mm Hg or other signs of fluid overload, stop fluid administration
RBC transfusion to maintain a hemoglobin ≥ 7 g/dL
Norepinephrine to maintain a MAP ≥ 65 mm Hg
Urine output ≥ 0.5 mL/kg
CRT ≤ 3 seconds

B. Fluid resuscitation protocol guided by advanced hemodynamic monitoring

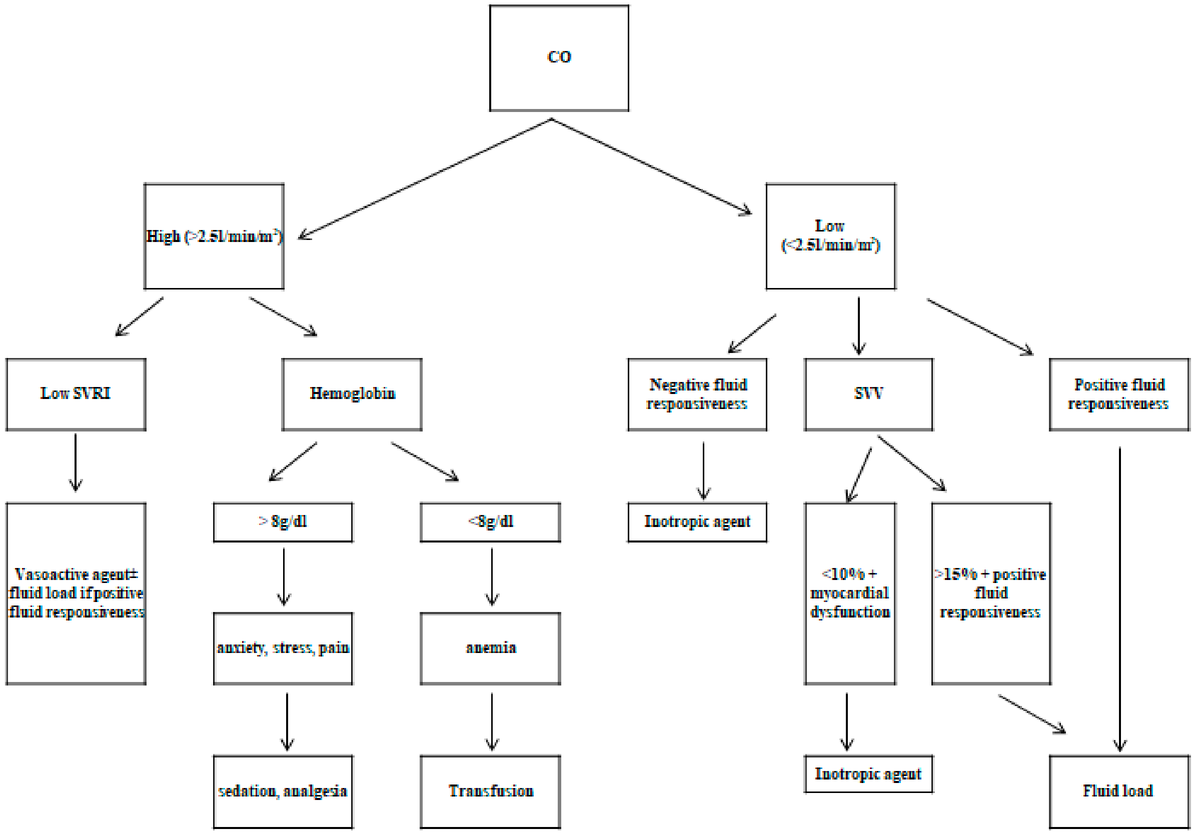

Supplemental Material 1. Fluid responsiveness was defined as an increase ≥ 15% in stroke volume (SV) at passive leg rising maneuver; CO: cardiac output; SVRI: systemic vascular resistance index; SVV: stroke volume variation.

Supplemental material 2: The hemodynamic parameters of the patients included in the study.

	Time Zero (T_0)	3rd H	*p* Value*	6th H	*p* Value**	24th H	*p* Value ***
Heart rate b/min Mean±SD	103.9±18.6	98.7±18.8	0.10	99.7±20.2	0.2	96.7±16.9	0.02
Systolic blood pressure mm Hg Mean±SD	118.0 ±21.0	122.7±18.1	0.15	127.3±17.8	0.005	130.5±17.9	0.0006
Diastolic blood pressure mm Hg Mean±SD	56.6±13.1	55.5±11.8	0.60	57.6 ±11.6	0.62	60.4±12.3	0.14
Mean arterial blood pressure mm Hg Mean±SD	75.2±13.6	74.8±11.6	0.86	78.4±12.1	0.14	81.2±13.4	0.009
Central venous pressure mm Hg Mean±SD	7.34±4.64	8.5±4.7	0.13	9.1±4.7	0.03	8.3±4.52	0.22
CI l/min Mean±SD	not monitored at time zero	3.5±0.7		3.6±1.01	0.3	3.27±0.68	0.75
SVI Mean±SD ml/m^2/beat)		35.7±10.7		37.2±12.5	0.44	33.1±8.6	0.45
GEDI Mean±SD ml/kg		647.5±166.7		632.5±219.6	0.79	605.1±120.6	0.16
ITBI Mean±SD ml/m^2		829.7±233.1		840.2±275.6	0.84	776.4±247.6	0.10
ELWI Mean±SD ml/kg		8.75±3.76		9.4±4.3	0.75	8.8±2.2	0.48
SVRI Mean±SD dynes-sec/cm–5/m2		1620.6±473.4		1609.9±495.8	0.86	1678.0±588.0	0.36
Norepinephrine Mean±SD mcg/kg/min	0.12±0.15	0.11±0.14	0.49	0.14±0.18	0.15	0.24±0.30	0.79
VDI Mean±SD	0.12±.15)	0.16±0.21	0.54	0.20±0.26	0.30	0.28±0.30	0.94
Creatinine Mean±SD µmol/l	218.3±192.7					200.7±174.2	0.45
Urea Mean±SD mmol/l	16.2±12.1					16.1±11.3	0.86
lactate (septic shock patients) Mean±SD mmol/l	3.9±2.5	3.8±3.0	0.30	3.8±3.1	0.30	2.3±2.8	< 0.0001
oliguria/anuria no of patients (%)	40 (56.3)	28 (39.4)	0.04	24 (33.8)	0.006	19 (26.7)	0.0003
Capilary refill time >3 sec no of patients (%)	17 (23.9))	12 (16.9)	0.29	8 (11.2)	0.04	4 (5.7)	0.002

Time 0 (T_0): time of study inclusion (ICU); 3rd, 6th, 24th hour: the transpulmonary thermodilution calibrations performed in Ev1000 Edwards Lifesciences® at these time frames; * comparison between time zero and 3rd hour;**comparison between time zero and 6th hour, except CI,SVI,GEDI, ITBI, ELWI (comparison with 3rd h) ITBI, ELWI (comparison with 3rd hour);*** comparison between time zero and 24th h, except CI,SVI,GEDI, ITBI, ELWI (comparison with 3rd h)
